# Reliability of the Function in Sitting Test (FIST)

**DOI:** 10.1155/2014/593280

**Published:** 2014-03-16

**Authors:** Sharon L. Gorman, Monica Rivera, Lise McCarthy

**Affiliations:** ^1^Department of Physical Therapy, Samuel Merritt University, 450 30th Street, Oakland, CA 94609, USA; ^2^McCarthy's Interactive Physical Therapy, 927 Vicente Street, San Francisco, CA 94116, USA

## Abstract

The function in sitting test (FIST) is a newly developed, performance-based measure examining deficits in seated postural control. The FIST has been shown to be internally consistent and valid in persons with neurological dysfunction but intra- and interrater reliability and test-retest reliability have not been previously described. Seven patients with chronic neurologic dysfunction were tested and videotaped performing the FIST on two consecutive days. Seventeen acute care and inpatient rehabilitation physical therapist raters scored six of the videotaped performance of the FIST on two occasions at least 2 weeks apart. Intraclass correlation coefficients were used to calculate the test-retest and intra- and interrater reliability of the FIST. ICC of 0.97 (95% CI 0.847–0.995) indicated excellent test-retest reliability of the FIST. Intra- and interrater reliability was also excellent with ICCs of 0.99 (95% CI 0.994–0.997) and 0.99 (95% CI 0.988–0.994), respectively. Physical therapists and other rehabilitation professionals can confidently use the FIST in a variety of clinical practice and research settings due to its favorable reliability characteristics. More studies are needed to describe the responsiveness and minimal clinically important level of change in FIST scores to further enhance clinical usefulness of this measure.

## 1. Introduction 

Research studies indicate that sitting balance ability is a substantial predictor of functional recovery after stroke [1, 2]. There is no universally accepted gold standard measure specific to sitting balance assessment, with many commonly used more global balance measures not specifically isolating sitting balance abilities or examining them at the International Classification of Functioning and Disability (ICF) level of impairment [3–5]. The function in sitting test (FIST) was designed as a concise test of functional sitting balance in patients following acute stroke [3] and another study supports the validity of the FIST in the inpatient rehabilitation population [6]. This test consists of 14 everyday functional tasks, quantifying sitting balance ability and describing sitting balance at the activity level of the ICF. Prior research has shown the FIST to have excellent internal reliability, as well as face, construct, content, and concurrent validity [3, 6]. Studies are underway examining the validity of the FIST in broader clinical populations along with investigating the concurrent and evaluative validity of the FIST in persons undergoing inpatient rehabilitation and the acute care neurological population [6, 7]. Additionally, with the increased emphasis on the use of valid and reliable outcome measures in persons with acute onset of neurologic disorders/diseases, there is need for measures that describe activity limitations in this population [3, 8–11].

Test-retest reliability is an important aspect of a measure, particularly when measuring constructs or performance that is not expected to change over time. In persons with chronic or stable diagnoses, there are limited changes in functional abilities and measures should demonstrate high test-retest reliability. In rehabilitation therapies, where documentation of functional changes is imperative, measures that detect changes during the rehabilitation process are a clear priority for clinical practice and the responsiveness of a measure is valuable. However test-retest reliability is still important as it describes the stability of a measure's score in a stable population. In order to properly examine the test-retest reliability of a measure, the use of a population with stable performance and a one to three day testing window between the two tests are commonly used to mitigate practice effects in the patients being retested [12].

The hallmark of a clinically useful test includes acceptable psychometric qualities related to the inter- and intrarater reliability of the measure [9, 13]. Intrarater reliability describes the ability of the same rater to obtain similar results when testing the same patient, while interrater reliability is related to the ability of multiple raters to arrive at the similar results on a particular measure in the same person. Determination of intra-rater reliability requires that patients to be retested after an interval by the same rater, while interrater reliability requires different raters score the same patient. Both are important in clinical and research settings, with measures that have high or excellent intra- and interrater reliability achieving increased acceptance for wide-spread use. Many design paradigms exist for the investigation of inter- and intratester reliability, but the most clinically applicable one is the partially standardized approach [14]. This methodology is achievable in clinical settings and requires standardized education methods of the raters but is not followed by rigorous checks or further assessment.

It can be difficult for performance-based clinical tests to have multiple raters scoring the same patient performance simultaneously when conducting interrater reliability research, so often these studies employ video recordings of patient performance. Video recording has been used for intertester reliability studies in numerous medical disciplines with acceptable results and can increase the participation of the raters, as it is easier to schedule rating sessions using performance recorded to video [15, 16]. To diminish the effects of recall in the raters, an interval of successive ratings of at least 2 weeks has been recommended [15, 17]. While no specific recommendations regarding the number of raters are prevalent in the literature, multiple studies of other balance tests used in rehabilitation included between seven [17] and ten therapist raters [18]. Additionally, a broad range of patient performance on the videos of the test is required, with the literature specific to other balance tests having between 4 and 20 patients whose performance is scored [17–19].

Currently, it is not known if the FIST has acceptable test-retest, intrarater, or interrater reliability. This study aims to close this gap in understanding of the psychometric qualities of the FIST, furthering its usefulness as a clinical and research measure of sitting balance function. The hypotheses to be tested in this study were that intertester reliability, intratester reliability, and the test-retest reliability of the FIST will fall within the “high” range with intraclass correlation coefficient between 0.70 and 0.89, as defined by Munro [20].

## 2. Methods

### 2.1. Participants

This study was approved by the Samuel Merritt University Institutional Review Board. Recruitment of all participants was voluntary and in compliance with the tenants of the Declaration of Helsinki, with all participants (or participants' legal representatives) signing informed consent forms. Participants fell into two categories: balance participants and therapist rater participants. Balance participants were purposively recruited from the community and included if they met the following criteria: (a) had a diagnosis of central nervous system neurological condition/disorder that was currently stable/chronic (no significant changes in function in the preceding 3 month period), (b) were over 18 years of age, (c) provided written informed consent by the participant or by proxy of a legally authorized representative, (d) scored 3 (moderate disability), 4 (moderately severe disability), or 5 (severe disability) on the Modified Rankin Scale, and (e) were proficient in speaking and reading in English. These balance participants were excluded if (a) medical condition(s) prevented testing procedures, such as but not limited to total hip arthroplasty due to restrictions of hip flexion range of motion, medical status such as subject not cleared for sitting/standing activities by physician, unstable angina, or orthostatic hypotension; (b) severe cognitive deficits limiting ability to follow simple directions; or (c) receiving any physical therapy intervention for balance deficits at the time of the study. Therapist rater participants were included in the study if they had (a) successfully completed the FIST online training module including posttest and/or a one-hour in-service training session, (b) a current license to practice physical therapy, and (c) proficiency speaking and reading in English.

### 2.2. Test-Retest Procedure

**Figure 1:**
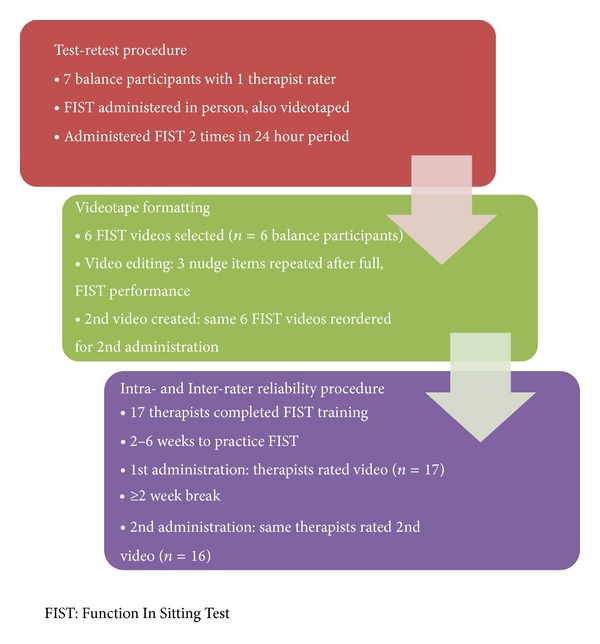
Study procedures.

Balance participants were recruited from the community from persons with stable or chronic neurologic conditions; this was to ensure that test-retest data would reflect participants' FIST scores that would be expected to be stable. All balance participants were tested and videotaped performing the FIST twice, one day apart, using the standard FIST testing protocol [3, 21]. Both sessions were videotaped and scored by the same one rater using the standard FIST score sheet (Table 1). A stable sample was purposively recruited inan attempt to control for any changes in FIST scores between the two sessions due to training or intervention effects [13, 14, 22]. Additionally, in an attempt to control the validity threats from maturation or history, the two scoring sessions were held only 1 day apart [13, 14, 22]. To attempt to control the therapist rater recall bias during the 2nd retest session, the therapist rater completed the initial FIST scoring sheets and did not have access to them until after the 2nd retest session was completed (24 hours later).  Figure 1 outlines all procedures for this study.

### 2.3. Intra- and Interrater Reliability Procedure

The videotaped performance of the balance participants' during the test-retest procedure was used for the reliability portion of this study. Balance participant videos were selected based on clear ability to view the administration of the FIST and to obtain a distinctive range of FIST scores. Six FIST test administrations of 6 participants' videos (1 video each) were edited into one longer video, approximately 40 minutes long. One balance participant did not consent to videotaping and could not be used in this portion of the study. The video with the clearest view of the balance participant's performance of the FIST (of the two sessions recorded) was selected. By using 6 different videos, a wider range of scores was available in attempt to make the results more generalizable. Videos were edited to replay the 3 nudge items after the full FIST video for each of the 6 balance participants to ensure the therapist raters viewed all 3 nudge FIST test items. This was needed because the 3 randomly performed nudge items on the FIST videos were occasionally missed by the therapist raters (e.g., rater looking down at score sheet during nudge and unable to score nudge) during the pilot testing of the videos being used to score the FIST. To decrease the effect of recall bias by the therapist raters during the second scoring session, these videos were then remixed into a second video package, randomizing the order of the 6 participants' appearance in the video [13–15, 19, 22, 23].

Seventeen therapist raters, purposively recruited from 3 different settings and 3 different facilities, completed the online FIST web-based training [21] or a one-hour training session prior to enrollment in the study and attended an in-service session to answer specific questions about participating in this study, including answering any FIST administration questions. This partial standardized approach to training was selected to allow therapists the opportunity to use the FIST after training with patients within their clinical practice to gain further familiarity with the FIST protocol [14]. Additionally, this training approach closely mimics how training occurs day to day in the clinical setting with regard to the administration of outcome measures. Approximately, two to six weeks after the introductory training, the first of two therapist rating sessions was held. After obtaining signed informed consent, basic demographic data about the therapist raters was collected. Afterwards, the balance participant videos were viewed by the therapist raters (*n* = 17), who scored the six FIST administrations using a standard FIST score sheet (Table 1). Therapist rater participants did not communicate with one another while scoring and only had the standard FIST score sheet to refer to during the scoring sessions. A second scoring session using the same protocol occurred at least 2 weeks later (*n* = 16) and used the same six balance participant videos but in a different order to further control any recall bias [14, 22].  Figure 1 outlines all procedures for this study.

### 2.4. Data Analysis

All analyses were conducted using SPSS version 21 [IBM, 2012]. Significance levels of 0.05 were used for all tests, and confidence intervals of 95% were also used. Two-way random model intraclass correlation coefficients (ICC_(2,1)_) were calculated using absolute agreement definitions.Test-retest reliability used the two scores on the FIST performed one day apart and scored by the same one rater, while intrarater reliability used the first session's FIST item scores (14 FIST items for each of the 6 videos, *n* = 16) and interrater reliability used the first and second scoring sessions FIST item scores (14 FIST items for each of the 6 videos, *n* = 17).

## 3. Results

Balance participants were primarily female (85.7%, *n* = 6) and had a mean age of 68.7 years. Medical diagnoses of the balance participants included Parkinson's disease (*n* = 1), multiple sclerosis (*n* = 1), and cerebrovascular accident (*n* = 5). Balance participants' performance reflected a variety of scores on the FIST, for individual FIST items as well as total FIST scores. Scores on individual items covered the full scoring range of 0 through 4, using the breadth of the FIST's scoring scale, and the overall FIST scores ranged from 11 to 56, out of the available 0–56.

Therapist rater demographics are presented using summary statistics in Table 2. ICC_(2,1)_ was excellent at 0.97 (95% CI 0.847–0.995) indicating excellent test-retest reliability of the FIST for use with both individuals in a clinical setting and/or groups in a research context [24]. ICC_(2,1)_ for intrarater reliability was calculated for the 16 therapist raters who scored the videos at the two time points and was found to be excellent (ICC = 0.99, 95% CI 0.991–0.995, *n* = 16). ICC_(2,1)_ for interrater reliability was also excellent (ICC = 0.991, 95% CI 0.988–0.994, *n* = 17). The SEM was 3.58 points (out of 56 possible points).

## 4. Discussion

### 4.1. Participant Demographics

Balance participants reflected a variety of scores on the FIST, for individual FIST items as well as total FIST scores, and represented neurologic conditions common in the population for which the FIST was created. Because of the small sample size of balance participants (*n* = 6 for inter- and intrarater reliability, *n* = 7 for test-retest reliability), it may be difficult to generalize these results to the broader range of potential patients without further study; however these preliminary results are encouraging.

Therapist rater demographics are presented in Table 2. The 17 therapist raters in this study closely resembled the gender representation for members of the American Physical Therapy Association, but this sample tended to be younger in age and have fewer years as a licensed physical therapist [25]. The therapist raters in this study were more likely to have a DPT degree both as their entry-level degree and as the highest degree earned [25]. However, the therapist raters participating in this study did cover a broad range of education and years in practice. Because the likelihood of using the FIST in clinical practice is higher in acute care and inpatient rehabilitation settings due to the fact that those populations exhibit more problems with sitting balance, and patients in these settings generally demonstrate a higher degree of sitting balance impairment, these therapist groups were purposively oversampled in this study compared to average US distribution, in attempts to make the results more generalizable [25]. While this study represents a small sample size of therapist raters (interrater *n* = 17, intrarater *n* = 16), high levels of reliability were still found even with a potentially underpowered study population.

### 4.2. Test-Retest Reliability

An ICC of 0.97 indicates excellent test-retest reliability of the FIST for use with both individuals in a clinical setting and groups in a research context [24]. Using balance participants with chronic histories that included stable neurological conditions and testing each participant twice with a 24-hour period ensured performance stability expectations. If a more acute balance participant population had been used, it would be more difficult to anticipate FIST score stability within the 24-hour testing window, and may have compromised the results of this study.

### 4.3. Intrarater and Interrater Reliability

This study demonstrates the excellent intra- and interrater reliability of the FIST. These results, when considered with previous research, indicate that the FIST is a reliable and valid measure of sitting balance function [3, 6]. Clinically, these results are valuable since intrarater reliability and interrater reliability are both in the excellent range; this is important in clinical practice as often the same therapist is not administering follow-up testing. Because many patients with sitting balance dysfunction transition through an episode of rehabilitation across multiple settings seeing multiple therapists, these findings indicate the FIST may be used confidently due to the excellent interrater reliability characteristics. Furthermore, the FIST has the potential to become a vital measure of tracking functional progress in persons who initially possess low function. Often these persons have an extended length of rehabilitation and must undergo serial measurements taken by different therapists in separate care settings. Jette et al. [11] cited common barriers to use of outcome measures by physical therapists, many of which related to the usefulness of the information gained to the specific patients seen: applicability of the measure to direct the plan of care, requirements for training, or costs associated with the use of the measure. All of these are not applicable to the FIST, as it is an activity-based measure appropriate for any patient with sitting balance dysfunction; the FIST test items are everyday functional tasks readily incorporated into the plan of care and are available at no cost [21]. As the results of this study have shown, minimal training is required to obtain excellent inter- and intrarater reliability. Additionally, as a performance-based measure, it addresses additional issues related to administration cited by Jette et al. [11].

These results are further strengthened given the training paradigm used in this study. Therapist raters were trained on FIST administration and scoring using an in-service training session or through a web-based training program. Training was then followed by a period of limited time to practice using the FIST on appropriate patients in their clinical work, followed by the opportunity to ask specific questions and/or to refer to the training website prior to data collection. This type of training most closely reflects the level of most clinical training opportunities in the administration of outcome measures, allowing study results to be more readily generalized to the larger population of therapists who may use the FIST. Additionally, this sequence represents a cost-effective training paradigm which is an important consideration in today's healthcare environment [11].

### 4.4. Limitations and Future Research

One limitation of this study was the use of only six balance participants to create the videos scored by the therapist raters. While every effort was made to use participants who demonstrated a wide variety of scores on all 14 FIST items, as well as to have a broad range of overall total FIST scores, not every possible score on each of the 14 items was included in this study. Generalizability was increased by using 17 physical therapist raters from three different settings and three different facilities; however results may not be able to be generalized to all possible raters, especially those from other healthcare professions who may use the FIST. Likewise, by providing training followed by an interval of at least 2 weeks, therapists were given the opportunity to practice administration of the FIST with their patients. However, some therapist raters in this study may have had more or less opportunity to use the FIST in clinical practice during this interval period which might have affected their familiarity when scoring the videos during the two scoring sessions. There was potential that the balance participants experienced a learning effect on the 2 FIST test administrations but is unlikely due to the short duration of the FIST (less than 8 minutes for all participants). Additionally, the test-retest reliability might have been affected by therapist rater recall bias, as the same rater scored all balance participants for these 2 sessions. While this study substantially adds to our understanding of the psychometric properties of the FIST, further psychometric studies of the FIST in different patient populations, determination of responsiveness of the FIST to change with rehabilitation interventions [6], and the ability of the FIST to predict fall risk and discharge disposition are still needed.

## 5. Conclusions 

The FIST is a reliable measure recommended for use in patients with neurological deficits to quantify deficits and/or abilities related to sitting balance. Overall, the FIST demonstrates excellent test-retest, intrarater, and interrater reliability with minimal training in administration. Rehabilitation clinicians and researchers can confidently use the FIST as a reliable and valid tool to measure activity-based deficits and outcomes related to sitting balance in both individual patients and for research purposes.

## Figures and Tables

**Table 1:** FIST scoring sheet. Complete details about FIST administration and scoring can be found online at http://www.samuelmerritt.edu/fist.

FIST item	Score
Anterior nudge: light pressure to superior sternum, no warning	
Posterior nudge: light pressure between scapular spines, no warning	
Lateral nudge: to dominant side/strong side, light pressure at acromion, no warning	
Static sitting: 30 seconds	
Sitting, nod “no”: left and right	
Sitting, eyes closed: 30 seconds	
Sitting, lift foot: dominant/strong side, lift foot 1 inch twice	
Pick up object from behind: object at midline, hands breadth posterior	
Forward reach: use dominant/strong arm, must complete full motion	
Lateral reach: use dominant/strong arm, lift opposite ischial tuberosity	
Pick object up from floor: from between feet	
Posterior scooting: move backward 2 inches	
Anterior scooting: move forward 2 inches	
Lateral scooting: move to dominant/strong side 2 inches	

Total	

FIST scoring: 4 independent: completes the task independently and successfully; 3 verbal cues or increased time: completes the task independently and successfully but may need verbal cues; 2 upper extremity support: unable to complete task without using upper extremities for support or assistance; 1 needs assistance: unable to complete task successfully without physical assistance; 0 complete assistance: requires complete physical assistance to perform task successfully and is unable to complete task successfully with physical assistance, or dependent.

**Table 2:** Demographics of therapist rater participants (*n* = 17).

Gender	Male 23.5% (*n* = 4)
	Female 76.5% (*n* = 13)

Age (mean)	34.5 years (SD = 10.8, range 24–64 years)

Mean FIST training time	55 minutes (range 45–60 minutes)

Years as licensed physical therapist (mean)	6.6 years (SD = 6.0, range 0–20 years)

Practice setting	Acute care hospital 29.4% (*n* = 5)
	Inpatient rehabilitation 58.8% (*n* = 10)
	Outpatient rehabilitation 5.9% (*n* = 1)
	Academic institution 5.9% (*n* = 1)

Facilities	Facility A 29.4% (*n* = 5)
	Facility B 64.7% (*n* = 11)
	Facility C 5.9% (*n* = 1)

Entry-level physical therapy degree	Bachelors 5.9% (*n* = 1)
	Masters 23.5% (*n* = 4)
	DPT 70.6% (*n* = 12)

Highest degree earned	Ph.D., E.dD., D.Sc. 11.8% (*n* = 2)
	DPT 64.7% (*n* = 11)
	Advanced masters (in PT) 5.9% (*n* = 1)
	Advanced masters (other) 5.9% (*n* = 1)
	None beyond entry-level 11.8% (*n* = 2)

SD: standard deviation; DPT: doctor of physical therapy.
